# Applying Prospect Theory to Participation in a CAPI/Web Panel Survey

**DOI:** 10.1093/poq/nfz030

**Published:** 2019-08-15

**Authors:** Peter Lynn

## Abstract

Prospect theory states that the influential power of avoiding negative outcomes is stronger than that of achieving positive outcomes. In a survey context, this theory has been tested with respect to not only participation in a CATI survey, but also giving consent to data linkage in CATI surveys. No study, however, has tested the theory with respect to participation in a CAPI or web survey. This study does so in a mixed-mode panel context; it also tests the moderating effects of time-in-panel, response history, and mode protocol. Results show that the framing of the survey participation request influences participation propensity in a way consistent with prospect theory, but only for relatively recent panel entrants. The opposite effect is found for long-term panel participants. No difference is found between mode protocols.

Prospect theory (Kahneman and Tversky 1979, 1984) is a general theory concerning the psychology of decision-making. It states that the influential power of avoiding negative outcomes is stronger than that of achieving positive outcomes. Experimental evidence is consistent with the theory in several contexts. For example, people are more willing to take actions to prevent a charity from losing $10 than they are to earn $10 for the charity (Kahneman 2011).

In a survey context, the theory has been applied to the decision to take part (or not) in a survey and to the decision to give consent (or not) to data linkage. Tourangeau and Ye (2009) carried out an experiment on a telephone follow-up to an RDD survey in the United States, in which interviewers emphasized either the positive benefits of participation or the negative consequences of not participating. They found a higher re-interview rate with the negative appeal. Two separate studies, also both on telephone surveys, found a higher rate of consent to data linkage with the negative wording. One of these studies took place in the United States (Kreuter, Sakshaug, and Tourangeau 2016), and the other in Germany (Sakshaug, Wolter, and Kreuter 2015). Bradburn (2016) has noted that the idea might also be applicable in other survey contexts.

This study applies prospect theory to a CAPI and web survey. Members of the Understanding Society Innovation Panel, a probability-based general population panel in the UK, were randomly allocated to one of two treatment groups. The control group received an advance letter that stressed the positive benefits of participation. (This is referred to as the control group, as this approach had been used in the advance letters for all previous waves of the Innovation Panel.) The treatment group instead received a letter that stressed the negative consequences of not participating, framed as a loss of value of the data that the respondent had already supplied at previous waves.

The primary research question is whether, in the context of a CAPI/web mixed-mode panel, the framing of the survey participation request can affect response rates. Specifically, a request emphasizing negative consequences of nonparticipation (negative framing) should induce higher response rates than a request emphasizing positive consequences of participation (positive framing).

However, in the panel context, the extent and nature of previous participation experience could moderate any effect of the framing of the participation request. In other words, the leverage of negative framing could differ between sample subgroups defined by previous participation experience (Groves et al. 2000). There are at least two mechanisms through which such a moderating effect could operate. The first is the psychological norm of consistency (Baumeister 1982), which states that people tend to respond to similar requests in a similar way. In a survey context, Groves, Cialdini, and Couper (1992) and Groves and Couper (1998) suggested that the norm may be invoked when people respond to survey participation requests. In a panel survey where most sample members have repeatedly participated in response to a positively framed request, a negatively framed request could be perceived as a different kind of request to the one that they are used to. In this circumstance, the sample members may feel less obliged to act consistently (than they did at previous waves of the survey). Thus, any positive effect of negative framing may be reduced or even reversed, the longer a sample member has been participating prior to the change in the framing of the participation request.

The second mechanism that could moderate any framing effect is the sunk-cost effect (Arkes and Blumer 1985). This refers to the tendency for decisions to be influenced by how much money, time, or effort has already been invested in a process. The greater the prior investment, the more likely it is that either further investment or risk-seeking behavior will be engaged in, and the less easy it will be to influence that future behavior. This suggests that, other things being equal, longer-term survey panel members should be less susceptible to any positive effects of the negative framing of the participation request (Thaler and Johnson 1990).

Given these two potential moderating mechanisms, the second research question is whether any framing effect on response rates depends on the extent and nature of previous participation in the panel.

The third research question concerns the role of primary data collection mode as a potential moderator. The written survey participation request differs substantially in nature between a web-first survey and a CAPI-first survey. In the web-first case, the recipient can act upon the invitation letter immediately by accessing the online questionnaire. In the CAPI-first case, the request forms part of an advance letter, which can only be actioned when the interviewer visits, typically some days or even weeks later. Furthermore, interaction with the interviewer might be a major influence on the participation decision, reducing the role of the advance letter. Therefore, the third research question is whether any framing effect on response rates depends on whether the request is an invitation to participate in a web survey or a CAPI survey, with the hypothesis being that any beneficial effects of negative framing should be stronger for a web survey.

## Methods

### DESIGN

A randomized experiment was carried out in wave 10 of the Understanding Society Innovation Panel, for which fieldwork was conducted between May 9 and October 11, 2017. The survey (Uhrig 2011; Jäckle et al. 2018) is based on a stratified random equal-probability sample of households resident in Great Britain, with an initial sample of 2,760 addresses at wave 1 of the survey in 2008 and an additional 960 addresses added at each of waves 4 (2011) and 7 (2014).^1^ All persons resident at those addresses at the time of the respective first wave of data collection are defined as sample members. At each subsequent wave, attempts are made to gain the cooperation of all sample members, whether or not they remain at the same address or with the same household members.

At each wave, all sample members aged 16 or over are asked to complete an individual interview (around 40 minutes) and one adult per household is additionally asked to complete a household interview (around 12 minutes). All sample members aged 10 to 15 are asked to complete a self-completion questionnaire (around 20 minutes). Questions regarding sample members 9 years old or younger are asked of a parent or guardian and in the household grid. People are withdrawn from the panel only if they adamantly refuse to continue or reside in a household in which no person has participated in either of the previous two waves. The panel is additionally depleted through death and emigration. The cumulative wave 10 response rate (percentage of still-eligible sampled persons participating in wave 10; AAPOR RR3) is estimated to be 19.7 percent for the original sample, 25.2 percent for the wave 4 refreshment sample, and 15.2 percent for the wave 7 refreshment sample.^2^

At wave 10, 3,521 sample persons were issued to the field. Due to a separate randomized experiment with mode protocols, approximately two-thirds were allocated to a mixed-mode protocol in which the first phase was an invitation to a web survey (“web-first”). The other one-third were administered a mixed-mode protocol in which the first phase involved face-to-face CAPI (“CAPI-first”). Web-first sample members who did not respond in the first phase were attempted by CAPI in a second phase and, in a final “mop-up” phase, were offered the option of a telephone interview. CAPI-first sample members were offered the options of telephone or web at the mop-up stage.^3^ Random allocation to mode protocol was carried out independently for each of the three samples (the original and the wave 4 and wave 7 refreshment samples).

At the start of fieldwork, each sample member aged 16 or over was mailed a letter. For the web-first sample, this constituted an invitation to complete the web survey and included the survey URL and a log-in code. For the CAPI-first sample, the letter constituted an advance letter informing the recipient that an interviewer would soon be visiting. Most of the content of the letter was otherwise identical for the two samples. Where possible, web-first sample members were additionally sent their letter by email, timed to arrive on the same day as the mail letter. The email version of the letter included a direct link to the questionnaire.

Additional new household members were identified in the field only after a household had been contacted. These persons are not part of the current experiment, as they were not sent the initial letter.

The initial letter included a paragraph designed to motivate sample members to participate by emphasizing the value of their data. This paragraph is the focus of the prospect theory experiment reported here. For a random half of sample members,^4^ this paragraph emphasized the additional value of participating again at the current wave. This is referred to as the “control group,” as this approach was taken at previous waves and on most surveys. For the other half (“treatment group”), the motivational paragraph emphasized the loss of value associated with not participating again. All persons within a household received the same treatment (i.e., the random allocation took place at the household level).

The control group received the positively-worded paragraph: “The information you have given us previously is very valuable and will become even more valuable if you participate again this year.” The treatment group received the negative wording: “The information you have given us previously is very valuable but will become much less valuable if you don’t participate again this year.”

As the experiment was designed to identify which form of wording was more effective at motivating existing panel members to continue participating, both experimental versions of the wording refer to the information given previously by the sample member, and the experiment was restricted to people who had previously participated at least once.

### DATA

Of the 3,521 sample persons issued to the field, 88 were new sample entrants who were therefore excluded from the experiment and 594 were children aged under 16 (so, not yet eligible for the individual interview). A further 38 had become ineligible (died or emigrated) by the time of fieldwork, leaving an analysis sample of 2,801.

Six variables are included in the analysis, all categorical. The dependent variable is OUTCOME, a dichotomous indicator of whether or not the individual interview was completed at wave 10. The key predictor variable is FRAMING, a dichotomous indicator of whether the sample member is in the treatment (negative request framing) or control group (positive request framing), as described above. The four moderator variables are TIME (time in sample: 9, 6, or 3 previous waves), PREVRESP (previous wave response status: respondent, nonrespondent, child), REGRESP (full interview at two-thirds or more of previous waves, fewer than two-thirds of waves), and MODE (survey mode: CAPI-first or web-first). TIME, PREVRESP, and REGRESP are intended to measure important aspects of previous participation experience. Descriptive statistics are presented in table 1.

**Table 1.** Descriptive statistics

Variable	*N*	%
OUTCOME		
Respondent at wave 10	1,960	70.0
Nonrespondent at wave 10	841	30.0
FRAMING		
Treatment (negative framing)	1,400	50.0
Control (positive framing)	1,401	50.0
PREVRESP		
Respondent at wave 9	2,101	75.0
Nonrespondent at wave 9	669	23.9
Child at wave 9	31	1.1
REGRESP		
Regular respondent	2,110	75.3
Irregular respondent	691	24.7
TIME		
9 previous waves	1,383	49.4
6 previous waves	633	22.6
3 previous waves	785	28.0
MODE		
CAPI-first	955	34.1
Web-first	1,846	65.9

The statistical significance of differences in response rates between treatment and control groups for each subsample was tested by means of *t*-statistics from logistic regression. The complex sample design was taken into account using a linearization approach to estimation with Stata’s SVY commands. All analysis was carried out in Stata version 15.1 using public data files (University of Essex 2018).

## Results

The treatment had no effect on the overall propensity to participate (table 2, first row). However, this absence of an overall mean effect masks heterogeneity in the effect dependent on the extent and nature of previous participation in the panel (table 2, rows 2 to 9). The treatment had a negative effect on participation propensity among original sample members, that is, those who had been asked to participate in nine previous survey waves (response rates 68.6 percent vs. 74.7 percent; *P* = 0.01). However, among the most recent sample, who had only been asked to participate in three previous survey waves, the effect was reversed, though marginally significant (response rates 68.4 percent vs. 63.3 percent; *P* = 0.064). Among the intermediate sample, with six previous waves, there was no effect. It is noteworthy also that the treatment had a negative effect among previous wave non-respondents (response rates 22.8 percent vs. 29.9 percent; *P* = 0.04), though not among irregular respondents (those who had completed the interview at fewer than two-thirds of survey waves).

**Table 2.** Response rate by treatment; total sample and subgroups; and Wald *P*-values from logit models

Sample subgroup	*n*	Response rate		*p*
		Framing: control	Framing: treatment	
Full sample	2,801	71.0	69.0	0.39
TIME				
Time in sample: 9 waves	1,383	74.7	68.6	0.01*
Time in sample: 6 waves	633	72.3	70.8	0.66
Time in sample: 3 waves	785	63.3	68.4	0.06
PREVRESP				
Previous wave respondents	2,101	84.1	83.4	0.72
Previous wave nonrespondents	669	29.9	22.8	0.03*
Previous wave children	31	61.5	88.9	0.09
REGRESP				
Regular respondent	2,110	79.5	77.8	0.48
Irregular respondent	691	44.8	42.4	0.53
MODE				
CAPI-first mixed-mode	955	67.1	67.4	0.94
Web-first mixed mode	1,846	73.0	69.8	0.28

Note.— *P*-values from a Wald test of the equivalence of coefficients in a logit model, estimated using test command in Stata 15.1. Full model results are included in part B of the online appendix.

*p < 0.05.

As regards MODE, the treatment did not have a significant overall effect in the context of either the web-first or CAPI-first protocols (table 2, rows 10 and 11).

Logistic regression modeling (results not shown) confirms the results of the bivariate analysis presented here. After testing main effects of treatment, time in sample, previous wave response status and mode, as well as all two- and three-way interactions involving treatment, the only significant predictors in the model are the two-way interactions between FRAMING and TIME and between FRAMING and PREVRESP (including main effects of both TIME and PREVRESP). The effects are in the same direction, and similar in magnitude, to the bivariate effects shown in table 2. For parsimony, only the bivariate results are presented.

## Discussion

With the standard gain-framing approach to respondent motivation (control), members of the most recent refreshment sample, who were being asked to take part for only the fourth time, were less likely to respond than other sample members (who were being asked to take part for the seventh or tenth time). With the loss-framing approach (treatment), this difference was not apparent. Consequently, loss-framing may have generated an improved response rate for the most recent panel recruits. However, loss-framing had the opposite effect for long-standing sample members, who were being asked to take part for the tenth time. This is an important finding, as it is the first time that negative framing has been shown to have a detrimental outcome. Researchers should be cautious about introducing such framing for loyal panel respondents.

The effect among longer-term panel members may relate to the norm of consistency and to sunk costs and panel loyalty. Loyal panel members, who have previously participated in the survey repeatedly in response to the standard gain-framing motivational statement, may feel offended that the value of participation is now being framed differently. The idea that all the data they supplied previously (their sunk costs) will be devalued if they do not participate again may feel a bit like coercion and may be perceived as something they should have been told earlier. This is of course speculation, but it is clear that the framing of the survey request impacted long-term panel members differently. The reasons merit further exploration, as a better understanding will help us better design survey requests for panel surveys. Further research could also usefully explore positive and negative wording of the participation request for new panel entrants (for whom reference to the value of the data they have previously provided is not relevant) and the effect of consistently negative framing at each wave (as the observed effect of negative framing for long-term panel members may be due to the change from positively framed requests to a negatively framed request). Meanwhile, the absence of any difference in effects between data collection modes might reassure us that the same approach can safely be used for both web and CAPI surveys.

## Supplementary Material

nfz030_suppl_Supplementary-Appendix

## Footnotes

1 Addresses were selected with equal probabilities from the Postcode Address File (Lynn and Taylor 1995), within 120 primary sampling units. The sample design is described in detail in Lynn (2009). A further sample of 1,415 addresses was added at wave 10 but was excluded from the experiment presented here, as the treatment required prior participation.

2 Details of the calculation appear in part A of the online appendix.

3 In the CAPI-first group, 92.6 percent of interviews took place in CAPI, 6.9 percent by web, and 0.4 percent by CATI; in the web-first group, 72.8 percent of interviews were by web, 25.5 percent by CAPI, and 0.3 percent by CATI.

4 Allocation was orthogonal to both mode protocol and sample origin, following the approach of Lynn and Jäckle (2019).

## References

[CIT0001] Arkes Hal R., and Blumer Catherine. 1985 “The Psychology of Sunk Cost.” Organizational Behavior and Human Decision Processes 35:124–40.

[CIT0002] Baumeister Roy F 1982 “A Self-Presentational View of Social Phenomena.” Psychological Bulletin 91:3–26.

[CIT0003] Bradburn Norman 2016 “Surveys as Social Interactions.” Journal of Survey Statistics and Methodology 4:94–109.

[CIT0004] Groves Robert M., Cialdini Robert B., and Couper Mick P.. 1992 “Understanding the Decision to Participate in a Survey.” Public Opinion Quarterly 56:475–95.

[CIT0005] Groves Robert M., and Couper Mick P.. 1998 Nonresponse in Household Interview Surveys. New York: Wiley.

[CIT0006] Groves Robert M., Singer Eleanor, and Corning Amy. 2000 “Leverage-Saliency Theory of Survey Participation: Description and an Illustration.” Public Opinion Quarterly 64:299–308. 11114270 10.1086/317990

[CIT0007] Jäckle Annette, Al Baghal Tarek, Burton Jonathan, Kaminska Olena, and Lynn Peter. 2018 *Understanding Society: The UK Household Longitudinal Study Innovation Panel, Waves* 1*–*10, *User Manual*. Colchester: University of Essex.

[CIT0008] Kahneman Daniel 2011 Thinking, Fast and Slow. London: Penguin.

[CIT0009] Kahneman Daniel, and Tversky Amos. 1979 “Prospect Theory: An Analysis of Decision Under Risk.” Econometrica 47:263–91.

[CIT0010] ———. 1984 “Choices, Values and Frames.” American Psychologist 39:341–50.

[CIT0011] Kreuter Frauke, Sakshaug Joseph W., and Tourangeau Roger. 2016 “The Framing of the Record Linkage Consent Question.” International Journal of Public Opinion Research 28:142–52.

[CIT0012] Lynn Peter 2009 “Sample Design for Understanding Society.” Understanding Society Working Paper 2009-01. Colchester: University of Essex.

[CIT0013] Lynn Peter, and Annette Jäckle 2019 “Mounting Multiple Experiments on Longitudinal Social Surveys: Design and Implementation Considerations.” In Experimental Methods in Survey Research: Techniques That Combine Random Sampling with Random Assignment, edited by Lavrakas Paul J., Traugott Michael W., Kennedy Courtney, Holbrook Allyson L., de Leeuw Edith D., and West Brady T. Hoboken, NJ: Wiley.

[CIT0014] Lynn Peter, and Taylor Bridget. 1995 “On the Bias and Variance of Samples of Individuals: A Comparison of the Electoral Registers and Postcode Address File as Sampling Frames.” Journal of the Royal Statistical Society Series D (The Statistician) 44:173–94.

[CIT0015] Sakshaug Joseph W., Wolter Sabine, and Kreuter Frauke. 2015 “Obtaining Record Linkage Consent: Results from a Wording Experiment in Germany.” Survey Methods: Insights from the Field. doi:10.13094/SMIF-2015-00012

[CIT0016] Thaler Richard H., and Johnson Eric J.. 1990 “Gambling with the House Money and Trying to Break Even: The Effects of Prior Outcomes on Risky Choice.” Management Science 36:643–60.

[CIT0017] Tourangeau Roger, and Ye Cong. 2009 “The Framing of the Survey Request and Panel Attrition.” Public Opinion Quarterly 73:338–48.

[CIT0017a] Uhrig S. C. Noah. 2011 “Using Experiments to Guide Decision Making in Understanding Society: Introducing the Innovation Panel.” In Understanding Society: Early Findings from the First Wave of the UK’s Household Longitudinal Study edited by S. McFall. Colchester: University of Essex.

[CIT0018] University of Essex, Institute for Social and Economic Research 2018 Understanding Society: Innovation Panel, Waves 1–10, 2008–2017, 9th ed. UK Data Service. SN: 6849. doi:10.5255/UKDA-SN-6849-10

