# Supplementary material for: Applying Prospect Theory to Participation in a CAPI/Web Panel Survey
Source: Public Opin Q. 2019 Aug 15;83(3):559–67. doi: 10.1093/poq/nfz030 (PMC6848831; doi:10.1093/poq/nfz030)
Supplement: nfz030_suppl_Supplementary-Appendix [file nfz030_suppl_supplementary-appendix.pdf]

APPLYING PROSPECT THEORY TO PARTICIPATION IN A CAPI/WEB PANEL SURVEY

Peter Lynn, University of Essex, UK

Online Appendix

A. Cumulative Wave 10 Response Rates

As described in section 4, the results presented in the paper are based on analysis of the 2,801 adult members of the three component samples who were issued to the field at wave 10 of the Understanding Society Innovation Panel. For illustrative purposes, section 3 presents the cumulative response rates for participation in wave 10. Out of the 2,801 issued sample members, 1,893 individual interviews were obtained at wave 10. The cumulative wave 10 response rates do not therefore reflect the proportion of the eligible sample included in the analysis base for the base, as the analysis base is not defined by participation at any particular wave. But they indicate levels of participation in the survey.

For each of the three samples, the cumulative response rate is estimated as the product of three components:

$$\widehat{RR} = \widehat{P_1} \times \widehat{P_2} \times \widehat{P_3}$$

where

$\widehat{P_1}$ is the estimated proportion of sample individuals whose household participated in the initial wave;

$\widehat{P_2}$ is the estimated proportion of individuals in participating households for whom an individual interview was completed at the initial wave; and

$\widehat{P_3}$ is the estimated proportion of individuals interviewed at the initial wave who were also interviewed at wave 10.

As the sampling frame does not include information on the number of residents at each address, the total number of individuals in the sample is unknown. The number of residents at each sample address is known only for the addresses where a successful household enumeration is carried out; not for (most) addresses where the household(s) did not respond. Therefore, P_1cannot be directly calculated. It must be estimated by first estimating the expected number of individuals in the sample of addresses. (This step circumvents the need to assume that household response rate is independent of household size, an assumption that would be needed if the observed proportion of households participating were used in the calculation instead of $\widehat{P_1}$.) The expected number of individuals in the sample is estimated as

$\hat{n} = a \sum_{i=1}^{7} i\pi_i$, where π_i is the proportion of households, according to the 2011 Census of Population, that contain i individuals (except π_7 , which indicates the proportion of households containing seven or more individuals) and a is the number of addresses in the selected sample, net of ineligible addresses (those at which no household resides). Thus, we estimate,

$\widehat{P_1} = {}^{m_1}/_{\hat{n}}$, where m_1 is the total number of persons in households that participated in the initial wave.

P_2 can be estimated in a more direct way, though note that only sample members aged 16 or over at the time of the respective survey wave are eligible for the individual interview, so the denominator is the total number of persons eligible for interview rather than the total number of sample members in sample households:

$\widehat{P_2} = {}^{m_3}/_{m_2}$, where m_2 is the number of persons aged 16 or over in participating (enumerated) households, and m_3 is the number of completed individual interviews.

For P_3, the denominator is the number of initial-wave respondents who are eligible to be interviewed again at wave 10. Thus, we should exclude initial-wave respondents who have either died or moved outside of the UK by the time of wave 10. The number expected to have died is estimated by applying age and gender-specific annual mortality rates, as published by the Office for National Statistics, to the age-gender distribution of the sample members interviewed at the initial wave. There is no reliable population information on the proportions of people who move abroad, so instead we exclude only the sample members who are known to have done so. This is likely to be a conservative approach as the survey is unlikely to have correctly identified all emigrants in the sample, so some will remain treated as non-respondents. Thus:

$\widehat{P_3} = {}^{m_4}\Big/_{(m_3 - \hat{d} - \hat{\imath})}$, where $\hat{d}$ is the number of initial wave respondents estimated to have died by wave 10, $\hat{\imath}$ is the number of initial wave respondents known to have emigrated by wave 10, and m_4 is the number of initial wave respondents who were interviewed also at wave 10.

Estimates of $\widehat{P_1}$, $\widehat{P_2}$, $\widehat{P_3}$, and their components are presented in table A1 for each sample.

Table A1: Response Rate Calculations

Sample	a	$\hat{n}$	m_1	$\widehat{\boldsymbol{P_1}} = {m_1}/{\hat{n}}$	m_2	m_3	$\widehat{\boldsymbol{P_2}} = {m_3}/{m_2}$	$\hat{d}$	i	m_4	$\widehat{\boldsymbol{P_3}} = {m_4}/{(m_3 - \hat{d} - i)}$	$\widehat{RR} = \widehat{\boldsymbol{P_1}} \times \widehat{\boldsymbol{P_2}} \times \widehat{\boldsymbol{P_3}}$
Wave 1 original	2,515	5,922	3,600	60.8	2,824	2,568	90.9	288	86	782	35.6	19.7
Wave 4 refreshment	853	2,008	1,136	56.6	893	777	87.0	48	19	373	51.2	25.2
Wave 7 refreshment	1,558	3,668	1,131	30.8	875	688	78.6	20	0	420	62.9	15.2

B. Logistic Regression Results

Here we present the full results of the models that underpin the P-values in table 2.

Table A2: Logistic Regression Models of Response Rate; Coefficients and Standard Errors

Sample subgroup	Constant		Framing		P (Adjusted Wald test)	N
	Coeff.	s.e.	Coeff.	s.e.		
Full sample	0.893	0.092	-0.093	0.106	0.386	2,801
Time in sample: 9 waves	1.380	0.193	-0.299	0.119	0.012	1,383
Time in sample: 6 waves	1.034	0.273	-0.075	0.172	0.664	633
Time in sample: 3 waves	0.323	0.230	0.224	0.148	0.065	785
Previous wave respondents	1.723	0.236	-0.055	0.151	0.719	2,101
Previous wave non-respondents	-0.487	0.268	-0.368	0.174	0.035	669
Previous wave children	-1.139	1.595	1.609	1.083	0.091	31
Regular respondent	1.454	0.228	-0.101	0.141	0.478	2,110
Irregular respondent	-0.112	0.256	-0.098	0.155	0.532	691
CAPI-first	0.699	0.282	0.013	0.176	0.942	955
Web-first	1.146	0.241	-0.153	0.141	0.282	1,846

Note: The dependent variable, survey outcome, is coded 1 for response and 0 for non-response. The predictor variable, framing, is coded 1 for the treatment (negative framing) and 0 for the control (positive framing). Thus, a positive coefficient for framing indicates that the treatment (negative framing) is associated with an increased tendency to respond.
